# The Physiology of Sleep

**Published:** 1907-08-03

**Authors:** Purves Stewart


### Notes on Current Neurology.

BY PURVES STEWART, M.D., F.R.C.P.

### THE PHYSIOLOGY OF SLEEP.

Kronthal has recently discussed this subject in an interesting and suggestive article.* The teaching of ordinary physiological text-books is somewhat misleading, inasmuch as too much stress is usually laid upon the suspension of cortical brain-function as being the essential element of sleep. Now, if this were all, an animal whose brain-cortex has been removed should no longer show the regular alternations between sleep and waking. Such, however, is not the case. In Goltz's well known experiments, a dog whose entire cerebrum had been removed, except a few traces at the base of the temporo-sphenoidal lobes, survived for a year and a half, and showed regular alternations of sleeping and waking just like an ordinary animal, though the periods were shorter than normal. Kronthal defines sleep as a transient state of the whole organism (not merely of the brain), during which most of the reflexes are diminished or abolished. Certain reflexes, such as those of respiration and cardiac action, persist during sleep, so also does the reflex tone of the sphincters. Sleep is a regularly recurring function of every living thing.

Several varieties of normal sleep can be recognised. Firstly, there is the sleep of exhaustion. This is not merely exhaustion of nervous tissues, but of the whole body. An excised muscle-fibre after prolonged stimulation loses its power of contracting unless it has an interval of rest, *i.e.*, of sleep, after which it regains that power. The same applies to every living cell. Sleep is essential for life. A dog will die if deprived of sleep for four or five days. On the other hand, an excised frog's muscle, if kept moist and at a suitable temperature, remains alive and capable of excitation for days at a time; but if it be constantly stimulated without intermission, it soon loses its excitability, passes into a doughy condition, and dies. Sleep, therefore, is not confined to the nervous system, but is shared by all the tissues of the body.

Secondly, there is toxic sleep, produced, for example, by substances such as morphia, chloroform, alcohol, etc. This is pathological, whilst the sleep of exhaustion is physiological. Toxic sleep differs from the sleep of exhaustion in that the organism can no longer be roused, even by the strongest stimuli. And here again narcotic poisons exercise their effects not merely on the nervous system but on the whole organism.

Another variety of sleep is that produced by cold. Artificial lowering of the temperature in guinea-pigs makes them drowsy. This variety of sleep has been attributed by some physiologists to cooling of the cortical cells of the brain. But all the cells of the body are affected. In this connection it is of interest to recall the phenomena of hibernation in certain animals, such as the hedgehog, dormouse, and bat, and in many reptiles and amphibians. Hibernation is simply the result of cold, since hibernating animals, if kept in a warm temperature throughout the winter, cease to hibernate. That a nervous system is not essential for hibernation was pointed out over sixty years ago by Barkow, who recalled the fact that plants hibernate in winter and wake again in spring. The sleep produced by cold resembles toxic sleep inasmuch as the falling asleep and awakening are much slower than in the normal sleep of exhaustion, and also in the fact that in "refrigeration sleep" stimuli cannot waken the subject; he only wakes when the temperature rises towards normal. If the temperature remains low, the sleep may pass on to death.

If deficient response to stimuli be an essential phenomenon of sleep, we should expect loss of reflex excitability to be necessary for the induction of sleep. Such loss of excitability may be brought about in several ways; by absence of stimuli, by non-sensitiveness of the sensory organs, and by interruption of sensory paths. Sleep from absence of stimuli is familiar; silence, darkness, absence of excitement all conduce to sleep. Secondly, if the sensory organs are deficient in reaction, stimuli no longer reach the organism. Sleep from deficient sensory perceptions is best exemplified in cases of total cutaneous anæsthesia. In such individuals, if the ears be filled with wool and the eyes closed, the patient falls asleep in a few seconds. Lastly, the interruption of the conducting paths of the central nervous system may produce sleep, even though, as we have already seen, the brain is not to be regarded as the essential organ affected; thus certain cerebral tumours and other intra-cranial lesions are associated with persistent drowsiness.

Somnambulism and the hypnotic trance must not be confounded with natural sleep. In these conditions the reflexes are not abolished, though they may show abnormal responses. They are to be regarded not as varieties of sleep, but rather as morbid mental conditions.

* "Neurologisches Centralblatt," 1907, p. 533.

